# The multifaceted role of m5C RNA methylation in digestive system tumorigenesis

**DOI:** 10.3389/fcell.2025.1533148

**Published:** 2025-03-06

**Authors:** Xinjun Hu, Yafeng Liu, Shujun Zhang, Kaijie Liu, Xinyu Gu

**Affiliations:** ^1^ Department of Infectious Diseases, The First Affiliated Hospital, College of Clinical Medicine, Henan University of Science and Technology, Luoyang, Henan, China; ^2^ Department of Oncology, The First Affiliated Hospital, College of Clinical Medicine, Henan University of Science and Technology, Luoyang, Henan, China

**Keywords:** RNA methylation, m5C modification, post-transcriptional regulation, biomarker, digestive system tumors

## Abstract

5-Methylcytosine (m5C) is a widespread RNA methylation modification, wherein a methyl group is enzymatically transferred to specific RNA sites by methyltransferases, such as the NSUN family and DNMT2. The m5C modification not only impacts RNA structure and stability but also governs post-transcriptional regulation by influencing RNA transport, translation, and protein interactions. Recently, the functional importance of m5C in complex diseases, including cancer, has gained substantial attention. Increasing evidence highlights the critical roles of m5C in digestive system malignancies, where it contributes to tumor progression by modulating oncogene expression and regulating processes such as tumor cell proliferation, migration, invasion, and resistance to chemotherapy. Furthermore, m5C’s involvement in non-coding RNAs reveals additional dimensions in elucidating their roles in cancer. This review summarizes recent advances in m5C RNA methylation research within digestive system tumors, focusing on its functional mechanisms, clinical significance, and potential applications. Specifically, it aims to explore m5C’s role in tumor diagnosis, prognosis, and treatment, while proposing future directions to address current challenges and broaden its clinical utility.

## 1 Introduction

RNA modifications have emerged as a pivotal aspect of epigenetics, drawing increasing attention in recent years (Barbieri and Kouzarides, 2020; Li G. et al., 2024; Orsolic et al., 2023). Various chemical alterations, including N6-methyladenosine (m6A), 5-methylcytosine (m5C), and N1-methyladenosine (m1A), play critical roles in regulating gene expression, RNA processing, stability, nuclear export, and translation (Orsolic et al., 2023; Roundtree et al., 2017; Shahrajabian and Sun, 2023; Zhang et al., 2023c). Aberrant RNA modifications are frequently implicated in the onset and progression of numerous diseases, particularly cancer, where they are key contributors to tumorigenesis, progression, and drug resistance (Han et al., 2023; Huang et al., 2020; Yang B. et al., 2021; Zhuang et al., 2023).

Among these modifications, m5C is a prominent methylation mark found in diverse RNA species (Sun et al., 2023; Wiener and Schwartz, 2021; Zheng et al., 2023). It is predominantly catalyzed by RNA methyltransferases, such as the NSUN family and DNMT2, and is commonly present in tRNA, rRNA, mRNA, as well as long non-coding RNAs (lncRNAs) and circular RNAs (circRNAs) (Cusenza et al., 2023; He et al., 2020; Nombela et al., 2021; Wang Y. et al., 2023). m5C serves multiple regulatory functions by modulating RNA structure, stability, nuclear export, and translation. It enhances RNA stability by preventing degradation and influences post-transcriptional processes, including splicing, transport, and translation (Squires et al., 2012). Recent advances in high-throughput sequencing technologies have gradually illuminated the role of m5C in cancer. Research suggests that m5C modification regulates oncogene expression and plays a role in controlling cancer cell proliferation, migration, invasion, and chemoresistance (Yang et al., 2017). Specifically, NSUN2-mediated m5C modification stabilizes oncogene mRNA, facilitating tumor progression (Chen S. J. et al., 2024). Furthermore, m5C’s involvement in non-coding RNAs, such as lncRNAs and circRNAs, has gained increasing recognition, as it modulates their function and protein interactions, thereby impacting cancer development and progression (Zheng et al., 2022).

In digestive system malignancies, dysregulated m5C expression is closely linked to tumorigenesis, progression, and patient prognosis (Lin and Kuang, 2024). Studies have revealed abnormal expression patterns of m5C methyltransferases, including NSUN2 and NSUN6, as well as m5C-binding proteins like YBX1 and ALYREF, in cancers such as esophageal, gastric, hepatocellular, colorectal, and pancreatic cancers. These alterations significantly affect tumor progression by regulating oncogene expression, tumor cell proliferation, migration, and responsiveness to chemotherapy. Consequently, investigating the role of m5C modifications in digestive system cancers is essential for deciphering the molecular mechanisms underlying tumor development and for devising novel diagnostic and therapeutic approaches. This review aims to comprehensively summarize the current advancements in m5C RNA methylation research within digestive system cancers, with a focus on its functional and molecular mechanisms across various tumor types, while exploring its potential as a biomarker for diagnosis, prognosis, and as a therapeutic target.

## 2 m5C RNA methylation

### 2.1 Tracing the origins: the discovery of m5C RNA methylation

The modification of RNA by m5C was first identified in the 1950s, when it was detected in tRNA and rRNA (Amos and Korn, 1958). However, only with the advent of high-throughput sequencing technologies did comprehensive investigations confirm the presence of m5C across a broader range of RNA species, including mRNA, miRNA, lncRNA, and circRNA (Amos and Korn, 1958; Li and Huang, 2024). In recent years, m5C has emerged as a pivotal epigenetic regulatory mechanism, garnering significant attention in cancer biology. Compared to other RNA modifications, m5C exhibits distinct and intricate patterns of distribution and function across various RNA types. Research has shown that m5C affects RNA structure and stability while playing a critical role in regulating gene expression and translation, thus contributing to cancer initiation and progression (Cusenza et al., 2023; He et al., 2020; Nombela et al., 2021; Squires et al., 2012; Wang Y. et al., 2023). Notably, the dynamic and adaptable nature of m5C modification underscores its significant role within the tumor microenvironment, potentially influencing cell fate decisions and cancer heterogeneity (Han et al., 2023; Yang B. et al., 2021; Zhang et al., 2021).

### 2.2 Advanced techniques for RNA m5C detection

With ongoing technological advancements, m5C RNA methylation detection methods have evolved significantly, moving from early quantitative techniques to modern, high-throughput approaches that allow precise localization (Motorin et al., 2010). One of the earliest methods, high-performance liquid chromatography (HPLC), facilitated the quantification of m5C content by separating and analyzing RNA fragments. While effective, HPLC’s limitations in identifying the exact location of m5C modifications have led to the development of more precise tools (Schaefer et al., 2009). Mass spectrometry (MS), recognized for its high sensitivity and accuracy, offers detailed insights into RNA molecules. By integrating efficient sample preparation with advanced analytical processes, MS generates refined maps of m5C modifications (Dominissini et al., 2012; Edelheit et al., 2013). As next-generation sequencing (NGS) technologies matured, chemical labeling approaches enabled more comprehensive detection. For example, bisulfite conversion combined with RNA-seq (BS-seq) selectively converts cytosine into uracil while preserving m5C, facilitating its identification in sequencing data (Cui et al., 2017; Frommer et al., 1992). Additionally, methylated RNA immunoprecipitation sequencing (MeRIP-seq) employs specific m5C antibodies to enrich m5C-modified RNA, significantly improving sensitivity and specificity (Zhang et al., 2024). At the forefront of technological innovation, single-molecule real-time sequencing (SMRT) has begun to reveal unique advantages (Ardui et al., 2018; Fang et al., 2012). SMRT enables direct RNA sequencing without requiring chemical labeling or immunoprecipitation, allowing the detection of various RNA modifications, including m5C, at the single-molecule level. Its ability to identify multiple RNA modifications simultaneously positions it as a promising tool in RNA modification research. These advanced detection techniques, particularly when employed in combination, provide higher resolution and sensitivity in m5C modification analysis, propelling further exploration in this rapidly advancing field.

### 2.3 Regulatory networks of m5C methylation

#### 2.3.1 Writers and eraser

**FIGURE 1:**
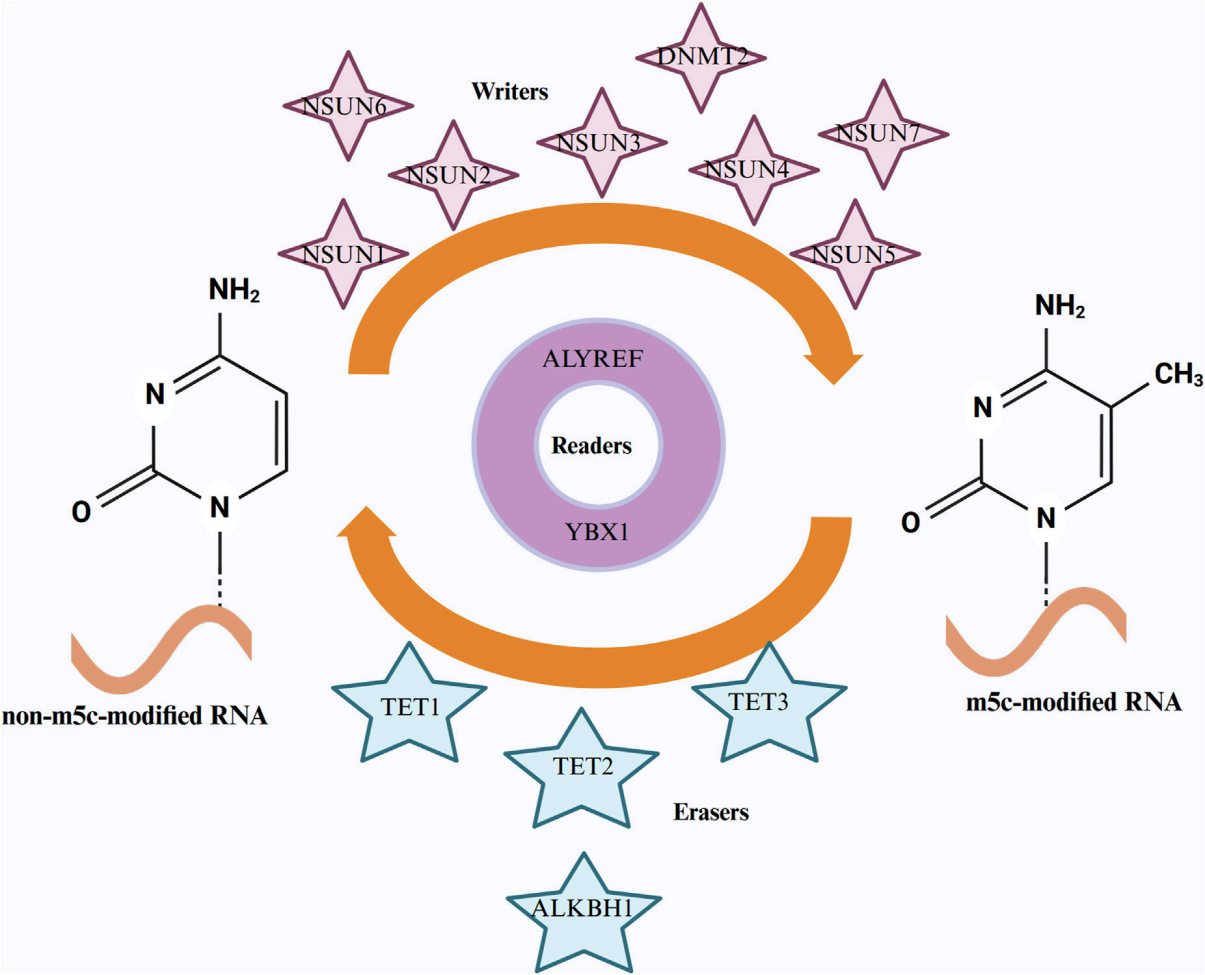
The regulatory mechanisms of RNA m5C methylation involving “Writers,” “Erasers,” and “Readers.” “Writers” are methyltransferases, such as the NSUN family members and DNMT2, responsible for adding m5C methyl groups to RNA. “Erasers,” including demethylases like the TET family and ALKBH1, remove these m5C modifications. “Readers,” such as ALYREF and YBX1, recognize and bind to m5C-modified RNA. Additionally, chemical structures of both unmodified RNA and m5C-modified RNA are depicted for comparison.

m5C modifications play an essential role in regulating various RNA molecules, with their functions intricately linked to specific regulatory factors (Chen et al., 2021). The primary m5C methyltransferases are members of the NSUN protein family (NSUN1-7) and DNMT2 (Figure 1) (Chen T. et al., 2023; He et al., 2020; Li et al., 2022; Sun et al., 2022) These enzymes catalyze m5C modifications at designated RNA sites, impacting a broad spectrum of biological functions. Each NSUN family member exhibits distinct functional specificity for different RNA types. For instance, NSUN2 is the predominant methyltransferase responsible for mRNA and tRNA methylation, whereas NSUN6 primarily modifies rRNA. DNMT2, initially identified as a DNA methyltransferase, was later recognized for its role in catalyzing m5C modifications in tRNA as well (Cheng et al., 2018; Li H. et al., 2024; Zhang et al., 2018).

The dynamic regulation of m5C modifications is not solely governed by methyltransferases but also relies on demethylases, which enable the reversibility and precise modulation of these modifications (Chen et al., 2021). The TET family of proteins (TET1, TET2, and TET3), originally characterized for their role in DNA demethylation, has been suggested to function in RNA demethylation as well, providing new perspectives on the plasticity of m5C modifications (Dusadeemeelap et al., 2022; Hu et al., 2013; Ito et al., 2011; Li et al., 2023b; Shen et al., 2021). In addition to the TET proteins, ALKBH1 (AlkB homolog 1), a key demethylase, has been shown to remove m5C modifications from RNA (Arguello et al., 2022). ALKBH1, a Fe(II)/α-ketoglutarate-dependent dioxygenase, was initially known for its role in demethylating N1-methyladenine and N3-methylcytosine in DNA (Zhong et al., 2024). More recent research has demonstrated that ALKBH1 can also demethylate m5C in RNA, particularly in tRNA and rRNA, where its activity is crucial for maintaining RNA stability and function (Arguello et al., 2022).

#### 2.3.2 Readers

Beyond the methyltransferases and demethylases, the biological effects of m5C modifications are further modulated by m5C-binding proteins (Wang N. et al., 2023). ALYREF (Aly/REF export factor) is a key m5C-binding protein that recognizes and binds to m5C-modified RNA, regulating processes such as RNA transport, nuclear export, and translation (Yang et al., 2017; Zhao et al., 2024). The interaction between ALYREF and m5C-modified RNA enhances both the stability and translational efficiency of the RNA, a function particularly critical in tumor cells (Klec et al., 2022; Nagy et al., 2021; Yang et al., 2023). Other m5C-binding proteins, including YBX1 and FMRP, also play vital roles in downstream regulation. YBX1 binds to m5C-modified mRNA, influencing its stability, splicing, and translation efficiency. In the context of cancer, YBX1 promotes tumor growth and metastasis by upregulating oncogene expression, and its overexpression is frequently linked to poor prognosis (Chen et al., 2019; Zou et al., 2024). Under stress conditions, YBX1 stabilizes mRNA, aiding tumor cells in surviving adverse environments (Liu X. et al., 2024; Meng H. et al., 2024; Wang et al., 2022). FMRP, primarily active in the nervous system, binds to m5C-modified RNA to regulate nuclear export and the localized translation of synaptic mRNAs (Chen Y. S. et al., 2023). The loss or dysfunction of FMRP is closely associated with neurodevelopmental disorders such as Fragile X syndrome, where it plays a pivotal role in maintaining neuronal function by regulating synaptic mRNA dynamics. These m5C-binding proteins perform multifaceted regulatory roles by interacting with m5C-modified RNA across various cellular contexts, contributing to disease development and progression (Chen Y. S. et al., 2023; Yang et al., 2022). Through the coordinated actions of methyltransferases, demethylases, and RNA-binding proteins (readers), the status of m5C modifications is finely tuned to meet specific physiological demands. This dynamic and reversible regulation ensures appropriate RNA stability, translational efficiency, transport, and RNA-protein interactions. The ability to tightly control m5C modifications is not only critical for normal cellular processes but also plays a significant role in cancer development and progression.

### 2.4 m5C-mediated regulatory dynamics across RNA classes

#### 2.4.1 Modulation of mRNA stability and translation

The intricate regulatory networks outlined above set the stage for understanding how m5C modifications impact specific RNA classes and their associated biological functions. The role of m5C modifications in mRNA has been extensively explored, revealing their critical impact on mRNA stability, nuclear export, and translation efficiency (Boo and Kim, 2020; Sun et al., 2023; Zhao et al., 2017). Research indicates that m5C modifications enhance mRNA stability by protecting it from ribonuclease-mediated degradation, thereby prolonging mRNA expression within cells (Selmi et al., 2021; Squires et al., 2012; Yang et al., 2022; Zhang et al., 2023b). NSUN2 introduces m5C modifications in the 5′UTR or 3′UTR regions of mRNA, which contributes to increased mRNA stability and augments translation efficiency (Chen S. J. et al., 2024; Yang et al., 2017). Furthermore, m5C modifications are intricately linked to mRNA nuclear export. Proteins such as ALYREF, which specifically bind to m5C-modified mRNA, facilitate its transport from the nucleus to the cytoplasm, thereby influencing gene expression regulation (Fan et al., 2019). These processes underscore the multifaceted regulatory functions of m5C modifications at the mRNA level, highlighting their pivotal role in the precise control of gene expression.

#### 2.4.2 Influence on non-coding RNA functions

In the realm of non-coding RNAs, m5C modifications also exert significant influence (Fabian and Sonenberg, 2012; Ferragut Cardoso et al., 2021; Xue et al., 2022). miRNAs, which are short non-coding RNAs involved in post-transcriptional gene regulation, are subject to regulation by m5C modifications. These modifications can affect miRNA precursor processing, thereby modulating the levels and activity of mature miRNAs, which in turn impacts critical cellular processes such as proliferation, differentiation, and apoptosis (Carissimi et al., 2021; Tang et al., 2023). Similarly, m5C modifications play a vital role in the regulation of lncRNAs (Ali and Grote, 2020; Herman et al., 2022). By influencing the stability and secondary structure of lncRNAs, m5C modifications enable lncRNAs to interact with proteins or DNA, thereby exerting control over downstream gene expression (Huang et al., 2023; Jiang et al., 2024; Pan et al., 2022). circRNAs, a unique class of non-coding RNAs characterized by their covalently closed circular structure, are also influenced by m5C modifications (Xue et al., 2021; Zhou et al., 2020). These modifications have been shown to affect both the biogenesis and functional roles of circRNAs. In the context of cancer, m5C-modified circRNAs are associated with promoting cancer cell proliferation, metastasis, and resistance to chemotherapy (Hou et al., 2024).

## 3 Role of m5C RNA methylation in digestive system cancers

### 3.1 Esophageal cancer

The elevated RNA m5C methylation observed in ESCC tumors stems from the overexpression of the m5C methyltransferase NSUN2 and the m5C “reader” Y-box-binding protein 1 (YBX1) (Liu L. et al., 2024; Niu et al., 2022; Su et al., 2021). Both NSUN2 and YBX1 are markedly upregulated in esophageal cancer tissues compared to adjacent normal tissues (Table 1). Higher NSUN2 expression is linked to more advanced cancer stages and heightened drug resistance, while elevated YBX1 expression correlates with poorer patient survival (Liu L. et al., 2024; Niu et al., 2022; Su et al., 2021). Functionally, NSUN2 overexpression significantly promotes cell proliferation, migration, and invasion (Table 2) (Liu L. et al., 2024; Niu et al., 2022; Su et al., 2021) *In vivo* experiments reveal that tumor growth and lung metastasis are markedly suppressed in NSUN2 knockout mice. NSUN2 also enhances resistance to irradiation *in vivo* (Niu et al., 2022). Moreover, YBX1 facilitates proliferation, invasion, and pluripotency maintenance in ESCC cells *in vitro*, increasing the sphere-forming ability of TE1 cells and regulating the expression of EMT and stem cell-associated proteins, including MMP1, MMP2, and β-catenin (Liu L. et al., 2024). YBX1 overexpression further promotes the growth and metastasis of esophageal cancer. Mechanistically, NSUN2 overexpression is positively regulated by E2F1, and NSUN2 induces m5C modification of growth factor receptor-bound protein 2 (GRB2), stabilizing its mRNA. LIN28B recognizes this modification, further stabilizing GRB2 mRNA (Su et al., 2021). Additionally, increased NSUN2 activity upregulates numerous oncogenes via m5C methylation, driving ESCC progression and the emergence of chemoradiotherapy resistance (Niu et al., 2022). YBX1, in an NSUN2- and m5C-dependent manner, binds to and stabilizes SMOX mRNA (Liu L. et al., 2024). The YBX1/m5C-SMOX axis accelerates ESCC progression by activating mTORC1 signaling.

**TABLE 1:** Expression profiles and associated clinical features of various RNA m5C modification regulators in digestive system cancers.

Type	RNA m5c regulator	Role	Clinical features	Refs
Esophageal cancer	NSUN2	Upregulated	Tumor stage and prognosis	Su et al. (2021)
Esophageal cancer	NSUN2	Upregulated	Drug resistance and prognosis	Niu et al. (2022)
Esophageal cancer	YBX1	Upregulated	Prognosis	Liu et al. (2024a)
Gastric cancer	NSUN2	Upregulated	Prognosis	Hu et al. (2021)
Gastric cancer	NSUN2	Upregulated	Prognosis	Fang et al. (2023)
Gastric cancer	NSUN2	Upregulated	Peritoneal metastasis and prognosis	Liu et al. (2023)
Gastric cancer	NSUN2	Upregulated	Degree of differentiation, lymph node metastasis, and Ki67 levels	Shen et al. (2024)
Liver cancer	NSUN1	Downregulated		Sun and Ding (2023)
Liver cancer	NSUN2	Upregulated	Prognosis	Song et al. (2023)
Liver cancer	NSUN4	Upregulated	Tumor stages, tumor grades, and prognosis	Cui et al. (2022)
Liver cancer	ALYREF	Upregulated	Tumor classification, TNM stage, tumor size, Ki67 level, and prognosis	Nulali et al. (2024)
Colorectal cancer	NSUN2	Upregulated	Prognosis	Zou et al. (2024)
Colorectal cancer	NSUN2	Upregulated	Tumor size and TNM stage	Chen et al. (2024b)
Colorectal cancer	NSUN6	Upregulated	Tumor stage and prognosis	Cui et al. (2024)
Pancreatic cancer	NSUN2	Upregulated	TNM stage, distant metastasis, and prognosis	Zhang et al. (2023b)
Pancreatic cancer	NSUN2	Upregulated	Prognosis	Chen et al. (2022)
Pancreatic cancer	ALYREF	Upregulated	Prognosis	Meng et al. (2024a)
Pancreatic cancer	NSUN6	Downregulated	T stage and prognosis	Yang et al. (2021b)

**TABLE 2:** Roles of various RNA m5C regulators and their associated genes in multiple digestive system cancers.

Type	RNA m5c regulator	Role	*In vitro*	*In vivo*	Related genes	Refs
Esophageal cancer	NSUN2	Carcinogenic effect	Cell proliferation, cell migration, and cell invasion	Tumor growth	E2F1, LIN28B, GRB2, PI3K, AKT, ERK, and MAPK	Su et al. (2021)
Esophageal cancer	NSUN2	Carcinogenic effect	Colony formation	Resistance to irradiation		Niu et al. (2022)
Esophageal cancer	NSUN2	Carcinogenic effect	Cell viability, cell migration, and cell invasion	Tumor growth and lung metastasis	NSUN2, SMOX, and mTORC1	Liu et al. (2024a)
Esophageal cancer	YBX1	Carcinogenic effect	Cell proliferation, cell invasion, and pluripotency maintenance	Tumor growth and metastasis	NSUN2, SMOX, and mTORC1	Liu et al. (2024b)
Gastric cancer	NSUN2	Carcinogenic effect	Cell proliferation, cell migration, and cell invasion		SUMO-2/3	Hu et al. (2021)
Gastric cancer	NSUN2	Carcinogenic effect	Cell proliferation		NR _ 033,928, IGF2BP3, and HUR	Fang et al. (2023)
Gastric cancer	NSUN2	Carcinogenic effect	Cell proliferation, cell migration, cell invasion, and peritoneal metastasis		AMPK, E2F1, YBX1, and ORAI2	Liu et al. (2023)
Gastric cancer	NSUN2	Carcinogenic effect	Cell migration and cell invasion		DIAPH2-AS1, and NTN1	Li et al. (2023b)
Gastric cancer	NSUN2	Carcinogenic effect	Cell proliferation	Tumor growth	CDKN1C	Mei et al. (2020)
Gastric cancer	NSUN2	Carcinogenic effect	Cell proliferation, cell cycle, cell apoptosis, and sensitivity to cisplatin and 5-fluorouracil		ERK1/2, Bcl-2, and Bax	Shen et al. (2024)
Liver cancer	NSUN1	Tumor suppressor	Cell proliferation, cell migration, and cell invasion		XPD	Sun and Ding (2023)
Liver cancer	NSUN2	Carcinogenic effect	Cell cycle arrest and cell sensitivity to sorafenib		Ras signaling pathway	Song et al. (2023)
Liver cancer	NSUN2	Carcinogenic effect	Cell proliferation, cell migration, cell invasion, and angiogenesis	Tumor growth	H19, G3BP1, and MYC	Sun et al. (2020)
Liver cancer	ALYREF	Carcinogenic effect	Cell proliferation and cell apoptosis	Tumor growth		Xue et al. (2023)
Liver cancer	ALYREF	Carcinogenic effect	Cell proliferation, cell migration, cell invasion, and EMT	Tumor growth and Ki67 levels	EGFR, and STAT3	Nulali et al. (2024)
Colorectal cancer	NSUN2	Carcinogenic effect	Cell proliferation, colony formation, and cell migration	Tumor growth	YBX1, SKIL, and TAZ	Zou et al. (2024)
Colorectal cancer	NSUN2	Carcinogenic effect	Cell proliferation, cell invasion, and stemness	Tumor growth and metastasis to the liver and lungs	H3K18la, YBX1, and ENO1	Chen et al. (2024a)
Colorectal cancer	NSUN6	Carcinogenic effect	Cell cycle and cell proliferation		METTL3	Cui et al. (2024)
Pancreatic cancer	NSUN2	Carcinogenic effect	Cell proliferation, colony formation, cell migration, cell invasion, and metastasis	Tumor growth, metastasis to the liver, lungs, intestines, and the formation of ascites	YBX1, and TIAM2	Zhang et al. (2023a)
Pancreatic cancer	NSUN2	Carcinogenic effect	Cell growth and drug resistance	Stromal fibrosis and ductal epithelial cell differentiation		Chen et al. (2022)
Pancreatic cancer	ALYREF	Carcinogenic effect	Cell proliferation, colony formation, and immune evasion	Tumor growth and Ki67 levels	JunD, SLC7A5, and mTORC1	Meng et al. (2024b)
Pancreatic cancer	NSUN6	Tumor suppressor	Cell proliferation and colony formation	Tumor growth and Ki67 levels		Yang et al. (2021a)

### 3.2 Gastric cancer

In gastric cancer (GC) tissues, NSUN2 is significantly upregulated compared to adjacent normal tissues, with its expression positively correlated to factors such as tumor differentiation, lymph node metastasis, elevated Ki67 levels, and peritoneal metastasis (Table 1) (Fang et al., 2023; Hu et al., 2021; Liu et al., 2023; Mei et al., 2020; Shen et al., 2024) Higher NSUN2 expression is associated with reduced overall survival (OS) in patients with GC, and univariate analysis identifies it as an independent prognostic risk factor for OS (Fang et al., 2023; Hu et al., 2021; Liu et al., 2023). Functionally, NSUN2 inhibition suppresses GC cell proliferation, migration, invasion, and peritoneal metastasis *in vitro*, while inducing cell cycle arrest and promoting apoptosis (Hu et al., 2021; Li et al., 2023a; Liu et al., 2023; Mei et al., 2020). Conversely, NSUN2 overexpression accelerates *in vivo* tumor growth, resulting in increased tumor volume and weight (Mei et al., 2020).

**FIGURE 2:**
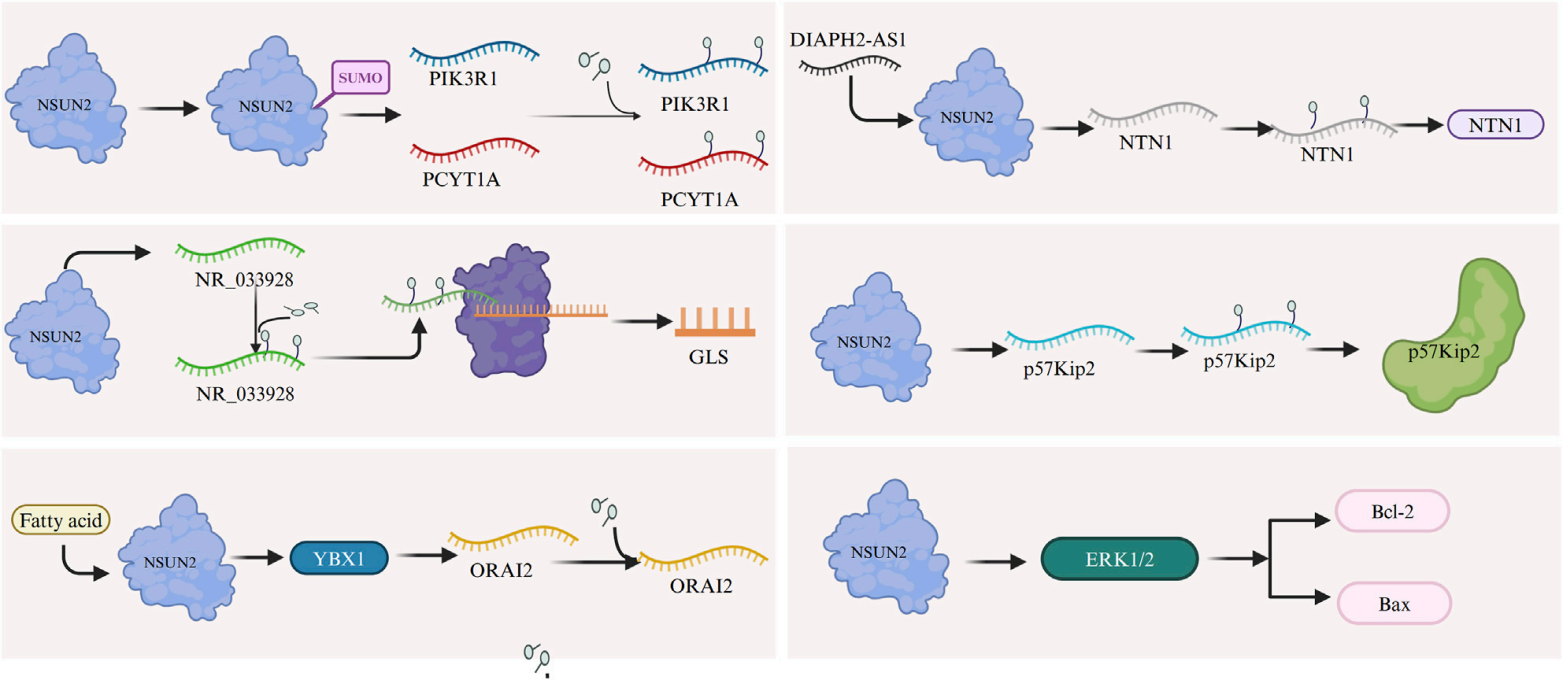
The m5C RNA methylation regulator NSUN2 promotes gastric cancer progression through several mechanisms. SUMO-2/3 interacts with and stabilizes NSUN2, facilitating its nuclear translocation and enhancing its oncogenic activity. NSUN2 methylates and stabilizes lncRNA NR_033928, driving cancer progression. E2F1 stimulates NSUN2 expression, while NSUN2 stabilizes ORAI2 mRNA via YBX1, increasing ORAI2 expression. DIAPH2-AS1 binds NSUN2, preventing its degradation through the ubiquitin-proteasome pathway, which enhances NSUN2 stability and upregulates NTN1 expression. NSUN2 may also inhibit CDKN1C (p57Kip2) through m5C modification, contributing to tumor progression. Furthermore, NSUN2 inhibition decreases ERK1/2 phosphorylation, reducing anti-apoptotic Bcl-2 levels and increasing pro-apoptotic Bax levels.

Mechanistically, the small ubiquitin-like modifier (SUMO)-2/3 directly interacts with and stabilizes NSUN2 and facilitates its nuclear translocation, enhancing its oncogenic function (Figure 2) (Hu et al., 2021) Additionally, the lncRNA NR_033928, methylated and upregulated by NSUN2 in an m5C-dependent manner, plays a critical role in promoting GC progression by enhancing its stability and expression (Fang et al., 2023). The transcription factor E2F1 further stimulates NSUN2 expression by binding to specific cis-regulatory elements (Liu et al., 2023). NSUN2-mediated m5C modification increases ORAI2 expression via YBX1-dependent stabilization of ORAI2 mRNA. Similarly, DIAPH2-AS1 binds to NSUN2, enhancing its stability by preventing ubiquitin-proteasome-mediated degradation (Li et al., 2023a). NSUN2 also promotes the upregulation of NTN1 through m5C modification and may facilitate GC progression by inhibiting CDKN1C (p57Kip2), a downstream target, in an m5C-dependent manner (Mei et al., 2020). Furthermore, NSUN2 inhibition reduces the phosphorylation of ERK1/2, leading to decreased levels of the anti-apoptotic protein Bcl-2 and increased levels of the pro-apoptotic protein Bax (Figure 2), thereby sensitizing GC cells to 5-FU/CDDP by enhancing apoptosis (Shen et al., 2024).

### 3.3 Liver cancer

In hepatocellular carcinoma (HCC) tissues and cells, NSUN2, NSUN4, and ALYREF are significantly upregulated, while NSUN1 is notably downregulated (Table 1) (Cui et al., 2022; Nulali et al., 2024; Song et al., 2023; Sun and Ding, 2023; Xue et al., 2023) ALYREF expression correlates positively with tumor classification, TNM stage, tumor size, and Ki67 levels, and elevated NSUN4 levels are indicative of more advanced tumor stages and grades (Cui et al., 2022; Nulali et al., 2024). Patients with higher expression levels of NSUN2, NSUN4, and ALYREF typically exhibit worse prognoses (Cui et al., 2022; Nulali et al., 2024; Song et al., 2023). ALYREF shows high diagnostic accuracy for HCC, with an AUC of 0.88, while NSUN4 expression serves as an independent prognostic risk factor (Cui et al., 2022; Nulali et al., 2024). Functionally, NSUN2 deficiency inhibits HCC cell proliferation, migration, invasion, and angiogenesis, while also increasing sensitivity to sorafenib (Song et al., 2023; Sun et al., 2020). Notably, chronic hepatitis B virus (HBV) infection is a key contributor to HCC. NSUN2 deficiency downregulates HBV expression, reducing HBV replication, whereas TET2 deficiency upregulates HBV expression (Feng et al., 2023). ALYREF further promotes proliferation, migration, invasion, and epithelial-mesenchymal transition (EMT) in HCC cells while suppressing apoptosis (Nulali et al., 2024; Xue et al., 2023). Silencing ALYREF significantly decreases tumor growth *in vivo* by reducing HCC cell proliferation.

Mechanistically, NSUN1 modulates XPD levels via m5C methylation, thereby inhibiting HCC progression (Sun and Ding, 2023). NSUN2 influences sorafenib resistance through the regulation of Ras pathway activity (Song et al., 2023). NSUN2-mediated RNA methylation promotes H19 lncRNA expression, with methylated H19 interacting with the oncoprotein G3BP1 to delay MYC mRNA decay, thereby driving tumor progression (Sun et al., 2020). Additionally, ALYREF recognizes the m5C modification of EGFR and regulates its levels, activating the STAT3 signaling pathway and further promoting HCC progression (Nulali et al., 2024).

### 3.4 Colorectal cancer

In colorectal cancer (CRC) tissues and cells, NSUN2 and NSUN6 are significantly upregulated (Chen B. et al., 2024; Cui et al., 2024; Zou et al., 2024). Specifically, NSUN2 expression is positively correlated with tumor size, TNM stage, and overall tumor stage, while NSUN6 expression is closely associated with ethnicity and tumor stage (Table 1) (Chen B. et al., 2024; Cui et al., 2024; Zou et al., 2024) Patients with CRC exhibiting elevated NSUN2 expression have poorer OS and disease-free survival (DFS) rates compared to those with lower NSUN2 levels (Chen B. et al., 2024; Zou et al., 2024). ROC curve analysis has highlighted NSUN6’s considerable diagnostic value across different cohorts (Tables 1, 2) (Cui et al., 2024) Silencing NSUN2 significantly reduces the stemness of CRC cells, and its knockdown effectively impairs tumor growth and metastasis to the liver and lungs *in vivo*. Furthermore, the NSUN2 inhibitor, Nsun2-i4, has demonstrated efficacy in significantly curbing tumor growth and reducing tumor burden in CRC (Zou et al., 2024). When combined with PD-1 therapy, Nsun2-i4 further amplifies tumor growth inhibition compared to monotherapy.

**FIGURE 3:**
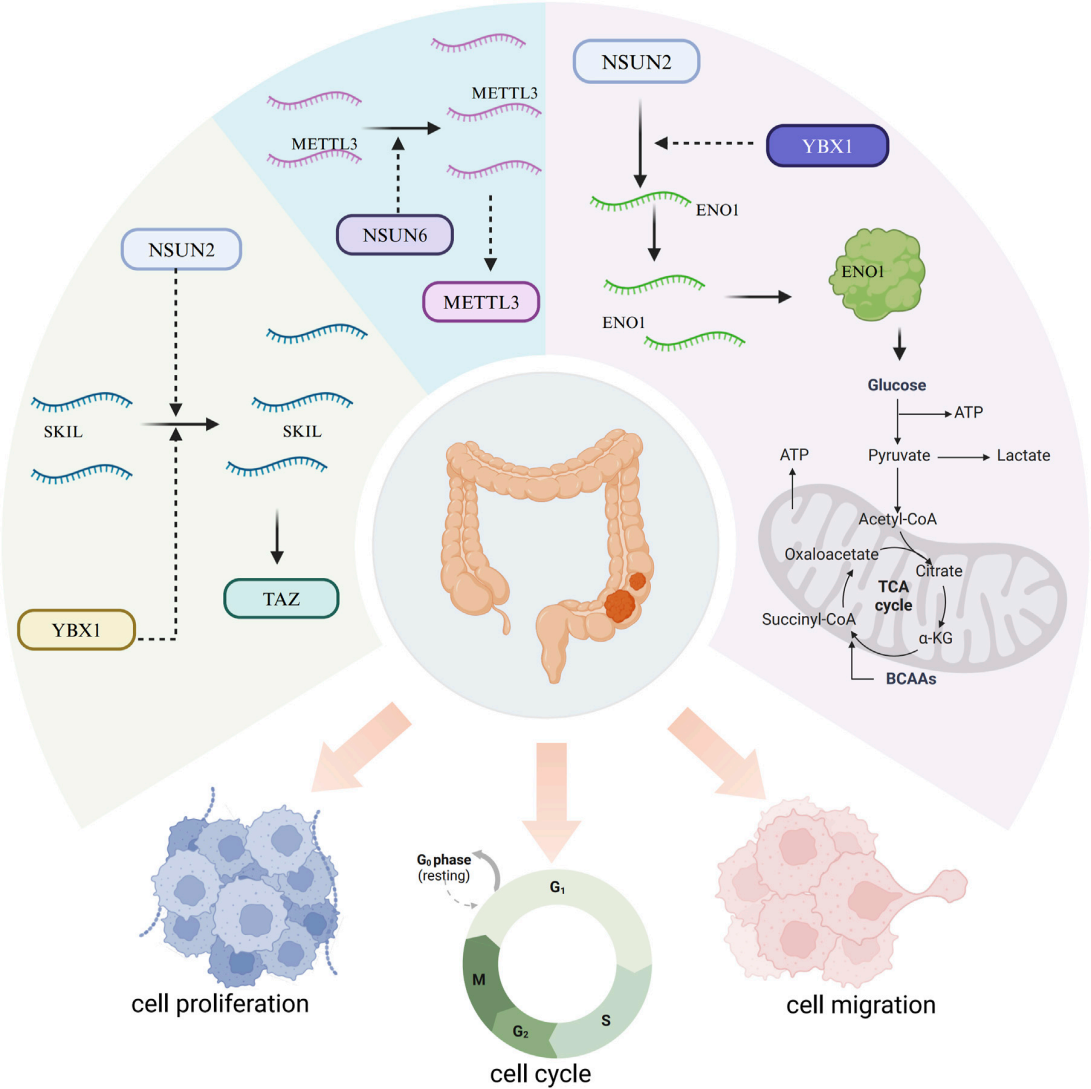
Mechanisms of m5C RNA methylation regulators in colorectal cancer. NSUN2 positively regulates the SKI-like oncogene (SKIL) via m5C modification in a YBX1-dependent manner, promoting tumor progression by activating TAZ. NSUN2 and YBX1 also collaboratively target Eno1 in an m5C-dependent manner, facilitating the reprogramming of glucose metabolism. NSUN6 knockdown reduces m5C modification on METTL3, resulting in METTL3 upregulation, which partially compensates for the cell cycle arrest and proliferation inhibition caused by NSUN6 knockdown.

Mechanistically, NSUN2 positively regulates the expression of the SKI-like oncogene (SKIL) through RNA m5C modification in a YBX1-dependent manner, thereby upregulating SKIL mRNA (Zou et al., 2024). Elevated SKIL expression promotes tumor progression by activating the transcriptional coactivator with PDZ-binding motif (TAZ) (Zou et al., 2024). Additionally, NSUN2 and YBX1 jointly target ENO1 in an m5C-dependent fashion, facilitating glucose metabolism reprogramming (Chen B. et al., 2024). Lactate produced by CRC cells enhances NSUN2 expression and its RNA-binding affinity through histone H3K18 lactylation (H3K18la), promoting m5C-mediated CRC progression and metastasis (Chen B. et al., 2024). Moreover, NSUN6 knockdown decreases m5C levels on METTL3, leading to METTL3 upregulation, which can partially offset the cell cycle arrest and proliferation inhibition triggered by NSUN6 knockdown (Figure 3) (Cui et al., 2024).

### 3.4 Pancreatic cancer

In pancreatic cancer tissues, NSUN2 and ALYREF are significantly upregulated, with NSUN2 expression positively correlating with TNM stage and distant metastasis (Table 1) (Chen et al., 2022; Meng Q. et al., 2024; Zhang G. et al., 2023) Elevated levels of NSUN2 and ALYREF are associated with shorter OS in patients with pancreatic cancer (Chen et al., 2022; Meng Q. et al., 2024; Zhang G. et al., 2023). In contrast, NSUN6 expression is reduced, and its levels are significantly correlated with the T stage and Ki67+ cell rate, showing a negative correlation with both OS and DFS (Yang R. et al., 2021). Regression analysis indicates that ALYREF and NSUN6 function as independent prognostic biomarkers for predicting pancreatic cancer outcomes (Meng Q. et al., 2024; Yang R. et al., 2021). Functionally, NSUN2 knockdown exerts a modest impact on pancreatic cancer cell growth and drug sensitivity, with effects becoming more pronounced over time (Chen et al., 2022). *In vivo*, NSUN2 knockdown leads to reduced stromal fibrosis and the restoration of ductal epithelial differentiation (Zhang G. et al., 2023). NSUN2 also promotes cell migration, invasion, and metastasis, and *in vivo* studies show that NSUN2 enhances tumor growth and metastasis to the liver, lungs, intestines, and ascites formation. Additionally, ALYREF has been shown to promote tumor growth and increase Ki-67 expression in tumor tissues, while NSUN6 inhibits pancreatic cancer cell proliferation and *in vivo* tumor growth (Meng Q. et al., 2024; Yang R. et al., 2021). Mechanistically, NSUN2-mediated m5C modification suppresses TIAM2 expression through YBX1, and disruption of the NSUN2/TIAM2 axis impairs the EMT, thereby slowing pancreatic cancer progression (Zhang G. et al., 2023). ALYREF, by enhancing CD8^+^ T cell functionality, contributes to the delay of pancreatic cancer development (Meng Q. et al., 2024). Furthermore, ALYREF directly regulates JunD in an m5C-dependent manner, leading to the transcriptional activation of SLC7A5. This activation of the JunD-SLC7A5-mTORC1 signaling pathway drives the proliferation of pancreatic ductal adenocarcinoma (PDAC) cells and facilitates tumor immune evasion (Meng Q. et al., 2024).

## 4 Clinical implications of m5C modification in digestive tumors

### 4.1 Diagnostic and prognostic value of m5C in digestive malignancies

m5C modifications have emerged as promising biomarkers for the diagnosis and prognosis of digestive system cancers. Research has established that the overexpression of m5C methyltransferases, including NSUN2, NSUN4, and NSUN6, as well as m5C “readers” such as YBX1 and ALYREF, is strongly linked to tumor progression, poor prognosis, and treatment resistance across cancers like esophageal squamous cell carcinoma (ESCC), GC, HCC, CRC, and PDAC. For instance, in ESCC, elevated NSUN2 levels are associated with advanced tumor stages and reduced patient survival, emphasizing its potential as a prognostic marker (Niu et al., 2022; Su et al., 2021). Similarly, in GC, NSUN2 overexpression is correlated with decreased overall survival and increased metastasis risk (Liu et al., 2023). In HCC, NSUN4 and ALYREF are independent prognostic indicators with high diagnostic accuracy (Cui et al., 2022; Nulali et al., 2024; Xue et al., 2023). NSUN6 plays a critical role in CRC and PDAC; in CRC, higher NSUN6 expression is linked to advanced tumor stages and lower survival rates, while in PDAC, reduced NSUN6 expression negatively correlates with prognostic markers like T stage and Ki67 positivity (Cui et al., 2024; Yang R. et al., 2021). ROC curve analysis further underscores NSUN6’s diagnostic significance across diverse populations, highlighting its potential as a biomarker for digestive system cancers (Yang R. et al., 2021). Despite its central importance in cancer biology, research on m5C faces technical challenges, such as the limited sensitivity and specificity of current detection methods and obstacles in translating these findings into clinical applications.

### 4.2 Therapeutic targeting of m5C methylation: a new frontier in cancer treatment

Targeting m5C modification pathways presents a promising therapeutic strategy for digestive system cancers (Wang C. et al., 2023). Inhibitors of m5C methyltransferases, such as NSUN2 inhibitors, have been shown to significantly reduce tumor growth and metastasis while enhancing the sensitivity of cancer cells to chemotherapeutic agents like cisplatin and 5-fluorouracil (Liu L. et al., 2024; Shen et al., 2024). In gastric and colorectal cancers, NSUN2 inhibition effectively suppresses cancer cell proliferation and migration, while improving the response to chemotherapy (Shen et al., 2024). NSUN6’s role in digestive tumors also suggests its potential as a therapeutic target. In PDAC, low NSUN6 expression is associated with enhanced cancer cell proliferation and decreased sensitivity to chemotherapy, whereas restoring NSUN6 activity inhibits tumor cell proliferation and reduces invasiveness, improving patient outcomes (Yang R. et al., 2021). In CRC, NSUN6 knockdown decreases m5C levels and upregulates METTL3 expression, which partially counteracts the cell cycle arrest and proliferation inhibition induced by NSUN6 knockdown (Cui et al., 2024). Additionally, targeting the interactions between m5C “readers” like YBX1 and ALYREF with m5C-modified RNA opens new avenues for disrupting key oncogenic pathways (Liu L. et al., 2024; Meng Q. et al., 2024). For example, inhibiting NSUN2 in PDAC reduces stromal fibrosis and restores ductal epithelial differentiation, thus slowing tumor progression and reinforcing NSUN2’s potential as a therapeutic target (Chen et al., 2022). Overall, targeting m5C modifications and their regulatory proteins offers substantial potential for the treatment of digestive system cancers and represents a critical avenue for advancing precision medicine strategies.

## 5 Conclusion and perspectives

As a vital epigenetic modification, m5C RNA methylation plays a pivotal role in the initiation, progression, and treatment of digestive system cancers. Studies have demonstrated that aberrant expression of m5C methyltransferases, such as NSUN2, NSUN4, and NSUN6, and m5C-binding proteins like YBX1 and ALYREF, is closely linked to the progression of various digestive tumors, including esophageal, gastric, hepatocellular, colorectal, and pancreatic cancers. Elevated levels of m5C modifications are frequently associated with advanced tumor stages, poor prognoses, and resistance to standard therapies, positioning m5C modifications as valuable biomarkers for cancer diagnosis and prognosis. On the therapeutic front, targeting m5C modifications and their regulatory proteins presents substantial promise. Inhibiting the activity or expression of m5C methyltransferases, such as NSUN2, can significantly suppress tumor cell proliferation, migration, and invasion while enhancing sensitivity to chemotherapy. Furthermore, disrupting the interaction between m5C-binding proteins, such as YBX1 and ALYREF, and m5C-modified RNA could interfere with key signaling pathways that drive tumor progression, offering new opportunities for the development of Therapeutic strategies targeting m5C modifications. Despite progress in understanding the role of m5C modifications in digestive system cancers, many aspects remain unexplored. Future research could focus on several critical areas: first, elucidating the dynamic regulatory mechanisms governing m5C modifications, especially the role of demethylases; second, investigating the tumor-specific effects of m5C modifications and their influence on tumor heterogeneity; and finally, developing more precise and effective m5C-targeted therapies aimed at improving clinical outcomes while minimizing adverse effects. These efforts will be crucial for unlocking the full potential of m5C modifications in cancer treatment.
